# Inhibition of glycolytic enzyme hexokinase II (HK2) suppresses lung tumor growth

**DOI:** 10.1186/s12935-016-0280-y

**Published:** 2016-02-16

**Authors:** Huanan Wang, Lei Wang, Yingjie Zhang, Ji Wang, Yibin Deng, Degui Lin

**Affiliations:** The Clinical Department, College of Veterinary Medicine, China Agricultural University, Beijing, 100193 China; Laboratory of Cancer Genetics, The University of Minnesota Hormel Institute, Austin, MN 55912 USA

**Keywords:** NSCLC, HK2, Kras, 2-DG, Apoptosis

## Abstract

**Background:**

The most common genetic changes identified in human NSCLC are Kras mutations (10–30 %) and p53 mutation or loss (50–70 %). Moreover, NSCLC with mutations in Kras and p53 poorly respond to current therapies, so we are trying to find a new target for the treatment strategies.

**Methods:**

Flow cytometry, crystal violet staining and immunoblotting were used to assess cell cycle arrest, proliferation and apoptosis in lung cancer cell lines after 2-DG treatment and lentivirus infection by shRNA knock down. IHC and western blotting were carried for NSG xenograft model with 2-DG treatment and lentivirus infection by shRNA knock down.

**Results:**

Knocking down Kras down-regulated the glycolytic enzyme hexokinase II (HK2) in KP2 (mouse lung cancer cell line with Kras mutation and p53 deletion) and H23 (human lung cancer cell line with Kras mutation and p53 mutation) cell lines. Genetic studies revealed that HK2 was required for the human and mouse lung cancer cell growth in vitro and in vivo. Our pharmacological studies confirmed that 2-DG, an inhibitor of HK2, inhibited human and mouse lung cancer cell growth through inducing cell apoptosis and autophagy.

**Conclusions:**

HK2 is a promising treatment target for NSCLC with Kras activating and p53 function loss.

## Background

More than one million people in the world die from lung cancer every year, so which is the leading cause of cancer mortality in human [1, 2]. Usually, lung cancer is defined in two types, small-cell lung cancer (SCLC) and non–small-cell lung cancer (NSCLC). NSCLC accounts for approximately 85 % of all the lung cancers at present. More than 70 % of NSCLC patients are advanced disease, and only 16 % can achieve the 5-year survival rate. For early stage, surgery combining with chemotherapy is the current standard of care. For stage III/IV of NSCLC, platinum-based combined chemotherapy is the standard approach, but chemotherapy has strong side effects to patients [3], so it is necessary to pursue new therapy strategies.

The most common mutations identified in human NSCLC are Kras mutations (10–30 %) and loss of function point mutations in p53 (50–70 %) [4]. Furthermore, for the patients harboring EGFR mutations (17 %) [5], resistant to EGFR tyrosine kinase inhibitors (TKIs) therapy, which has been proposed that the mutations in Kras and loss of functions in p53 may be the mechanism of primary resistance to EGFR TKI [6, 7]. And many researches demonstrated that the lung cancer patients, harboring mutations in Kras or loss of functions in p53, have poor clinical outcomes to chemotherapy and EGFR TKIs [8–10]. Therefore, inhibition of Kras expression or stimulation of p53 functions is attractive therapeutic strategy for this disease. However, it has, so far, been unsuccessful to attempt to develop drugs that target oncogenic Kras and convert mutant p53 proteins to a functional state [11–13], so we try to find Kras and p53 downstream therapeutic targets.

The well-established Kras gene encodes a small GTP-binding protein that serves vital roles not only in mediating cell growth, differentiation and apoptosis but also in regulating cell metabolism [14, 15]. Oncogenic Kras causes mitochondrial metabolism and the generation of reactive oxygen species (ROS) through regulation of the ERK-MAPK signaling pathway [16]. And it also mediates cancer metabolism by stimulation of glucose uptake and driving glucose intermediates into pentose phosphate pathways (PPP) and the hexosamine biosynthesis [15]. Activation of oncogenic Kras leads to mitochondrial dysfunction, causing decreased respiration, and increased glycolysis [17]. Therefore, oncogenic Kras might promote and maintain tumor growth by increasing the Warburg effect and anabolic (biosynthesis) pathways.

Hexokinases (HKs) catalyze the first essential step in glucose metabolism by phosphorylation of glucose to glucose-6-phosphate (G-6-P) [18]. There are four major isoforms (HK1, HK2, HK3, and HK4) characterized in mammalian tissue [19]. Among these, only the high level of HK2 expression has wildly been observed in cancer cells and is associated with poor overall survival in cancer patients [20, 21]. Patra et al. [18] found HK2 overexpression in mutant Kras overexpression and p53 knock out transgenic mouse models. Now our genetic studies in details address that HK2 is required for lung cancer cell growth in mouse KP2 cell (mouse lung cancer cell line with Kras mutation and p53 deletion) and human H23 cell (Human lung cancer cell line with Kras mutation and p53 mutation) in vitro and in vivo. And our pharmacological studies furthermore suggest that HK2 is one of the most important potential therapy targets for Kras overexpression and p53 function lose-driven lung cancer.

## Methods

### Cell lines, cell culture and reagents

Mouse lung cancer cell (KP2) line was generous gifted from Prof. Taylor Jackson. Human lung cancer cell (H23) was obtained from American Type Culture Collection. KP2 and H23 cells stably expressing GFP-LC3. All these cells were cultured in DMEM supplemented with 10 % FBS. 2-DG was purchased from Sigma-Aldrich and formulated in PBS.

### Plasmids and viral transfections

PLZW plasmids expressing human HK2 were obtained from Addgene. All other shRNAs were from the BioMedical Genomics Center at The University of Minnesota. These are lentiviral shRNAs and are as follows: Kras (TRCN0000034384 and TRCN0000055356; TRCN0000055357 for human and mouse), HK2 (TRCN0000037670 and TRCN0000037609; TRCN0000037669 for human; and TRCN0000037672 for mouse), AKT (TRCN0000039793; TRCN0000039794; TRCN0000039796 for human and mouse). In this study, we used the calcium phosphate transfection method to transfect vector into actively growing HEK-293T cells as described previously [22]. In briefly, the pLKO.1 vector backbone as the negative control vector has no hairpin insert. Firstly, shRNA-encoding plasmids need to be mixed thoroughly with envelope and packaging plasmids (VSVG, REV and pMDL) and then co-transfected into adherent HEK-293T cells using the calcium phosphate method. Collected virus-containing supernatant at 36 h after transfection, and centrifuged to remove cell and cell debris, and then infected the target cell with 8 μg/ml polybrene. To generate stable cell lines, cells were selected with 8 μg/ml puromycin 24 h later and knockdown efficiency was detected by immunoblotting.

### Cell growth evaluation and clonogenic survival assay

Seeded 2 × 10^5^ cells in each well in 6-well plates to analyze cell growth by 2-DG treatment. Incubated the cells overnight for cells attaching. To study whether the inhibition of cell growth treated with 2-DG is dose and time dependent, we collected cells treated with different concentration of 2-DG (2.5, 5,10, 10 mg/ml) for 48 h and cells treated with 10 mg/ml 2-DG for different time (12, 24, 48, 60, 72 h). Stained the cells with 0.25 % (w/v) trypan blue and counted. Washed the cells twice with PBS, and fixed in 10 % formalin for 10 min at room temperature, and then stained with methanol (10 % v/v) containing crystal violet (0.1 % w/v). Removed excess crystal violet and washed several times with distilled water and dried them. For clonogenic survival assay, seeded 2 × 10^4^ cells in each well of 6-well plate with stably expressing shRNA. After 1 week, fixed cell colonies in 10 % formalin and stained with crystal violet (0.1 % w/v).

### Western blotting

Washed the cells twice with ice-cold PBS and then lysed with ice-cold lysis buffer (50 mM HEPES pH 7.4, 150 mM NaCl, 2 mM MgCl2, 5 mM EGTA pH 8.0, 1 mM dithiothreitol, 0.5 % Triton X–100, 10 % glycerol, 1 mM Na3VO4, 1 μM microcystin–LR and protease/phosphatase inhibitor cocktail) for 30 min on ice. The lysates were centrifuged at 12,000 rpm for 5 min at 4 °C. Equivalent samples were resolved by SDS-PAGE and transferred onto PVDF membranes. Membranes were blocked with 5 % non-fat milk in PBS and then probed with indicated primary antibody overnight at 4 °C. In this study, the following antibodies were used: Kras (Millipore), HK2 (Cell signaling technology), Cleaved PARP (human) (Cell signaling technology), Cleaved PARP (mouse) (Cell signaling technology), LC3II (Cell signaling technology), Alpha-tubulin (Santa Cruz Technologies) and all of them were used at 1:1000 dilution. Then, primary antibody was detected with HRP-conjugated anti-mouse or anti-rabbit secondary antibody (GE Healthcare). Western HRP substrate was from Millipore.

### Immunohistochemistry (IHC)

All immunohistochemical analyses were carried out as previously described [23]. In this study, the following antibodies were used: LC3II, cleaved caspase-3 (Cell Signaling) and Ki67 (Millipore), and all of them were used at 1:100 dilution.

### Xenograft mouse model and treatment

The animal protocol was approved by the Institutional Animal Care and Use Committee of the University of Minnesota, and carried out at the Hormel Institute’s AAALAC-accredited animal facility. Subcutaneously injected 1 × 10^6^ cells (suspended in 100 μl of PBS) into the lower flank of NSG mice (005557 from The Jackson Laboratory; http://jaxmice.jax.org/strain/005557.html). To further study the Kras and HK2 function in vivo, the mice were injected with control, Kras knockdown KP2 cells, HK2 knockdown KP2 cells, or rescuing HK2 KP2 cells, monitored for tumor progress and euthanized all mice at 4 weeks after injection. Tumors were weighed and photographed. Once xenograft tumors for the study of 2-DG treatment were established (the tumor volume is about 50–100 mm^3^), two groups of mice were treated with PBS (Control) or 2-DG (800 mg/kg in PBS) by I.P. injection (daily for 15 days). Mice were euthanized, and tumors were dissected, weighed and fixed in 10 % formalin for histopathology and IHC analysis.

### Statistical analyses

Statistical significance is determined by Student’s t test or ANOVA analysis with Graphad Prism (v.5). P < 0.05 is considered to be statistically difference, and P < 0.01 is significant difference.

## Results

### Knockdown of oncogene Kras suppresses lung cancer cell growth and down regulates HK2 expression in vitro and in vivo

**Fig. 1:**
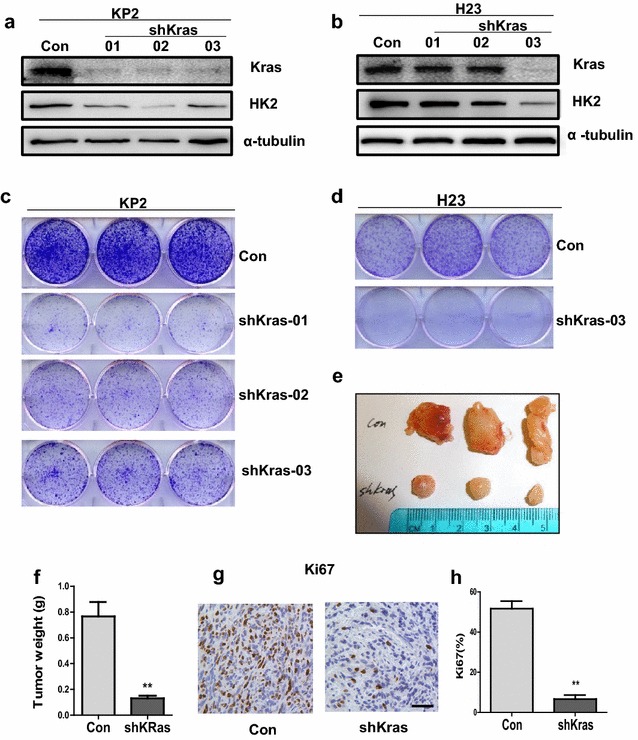
shRNA-mediated knockdown of oncogenic Kras reduces HK2 expression and suppresses growth in NSCLC cell lines. **a**, **b** Protein level of Kras and HK2 was detected in KP2 and H23 cells expressing shRNAs for Kras. **c**, **d** Clonogenic survival assays were performed to assess cell growth. Colonies were stained by *crystal violet* after 7 days of cell growth. **e**, **f** Xenograft tumor growth. 1 × 10^6^ cells expressing scramble shRNA (CON) or shRNA for Kras were subcutaneously injected to the lower flank of NSG mice. Representative images of tumors at 4 weeks after injection are shown (**e**). Quantification of tumors weight (P < 0.01) (**f**). **g** IHC staining of cell proliferation marker Ki67 from control or Kras-knockdown KP2 cells. **h** Quantification of Ki67 expression from representative images shown in **g** (P < 0.01)

Kras expression is very important in Kras-driven lung cancer cell lines. In this study, we chose KP2 and H23 cell lines to confirm the essential role of oncogenic Kras in Kras-driven lung cancer. First we knocked down oncogenic Kras expression in KP2 and H23 cell lines with Lentivirus-mediated small hairpin RNA targeting Kras caused significant reduction of Kras (Fig. 1a, b). Then we generated three stable cell lines in KP2 cells and one stable cell line in H23 cells with varying degrees of Kras protein reduction. ShKras-01, shKras-02 and shKras-03 can robustly decrease total Kras protein in KP2 cells compared with control vector-infected cells. However, only shKRAS-03 can robustly reduce Kras protein in human H23 cells, and shKras-01and shKras-02 cannot decrease Kras. Knockdown of Kras in KP2 cells and H23 cells significantly attenuated their colony formation by crystal violet staining analysis (Fig. 1c, d). Furthermore, the growth of KP2 xenograft tumor cells stably expressing shKras in NSG mice was significantly suppressed compared to KP2 cells with a vector shRNA (P = 0.0028) (Fig. 1e, f). IHC staining of ki67 shows tumor cell growth was obviously inhibited in Kras-knockdown KP2 xenograft group (P = 0.001) (Fig. 1g, h). These data support that shRNA-mediated knockdown of oncogenic Kras suppresses growth of KP2 cells and H23 cells in vitro and in vivo.

Although oncogenic Kras is one of the most important of the potential targets for drug development, so far, it has been unsuccessful to attempt to develop drugs that directly target oncogenic Kras. Therefore, it is very important for Kras-driven lung cancer therapy to pursue the key gene regulated by Kras, and to find its inhibitors or drugs. To decipher the relations of Kras and HK2, we checked HK2 protein by immunoblotting in Kras-knockdown cells, KP2 and H23. As shown in Fig. 1a, HK2 is significantly decreased in Kras-knockdown KP2 and H23 cells compared with the mock group. HK2 protein level had no significant change when shKRAS-01 and shKRAS-02 did not knock down Kras protein in H23 cells in Fig. 1b.

### HK2 is required for lung cancer cell growth in vitro and in vivo

**Fig. 2:**
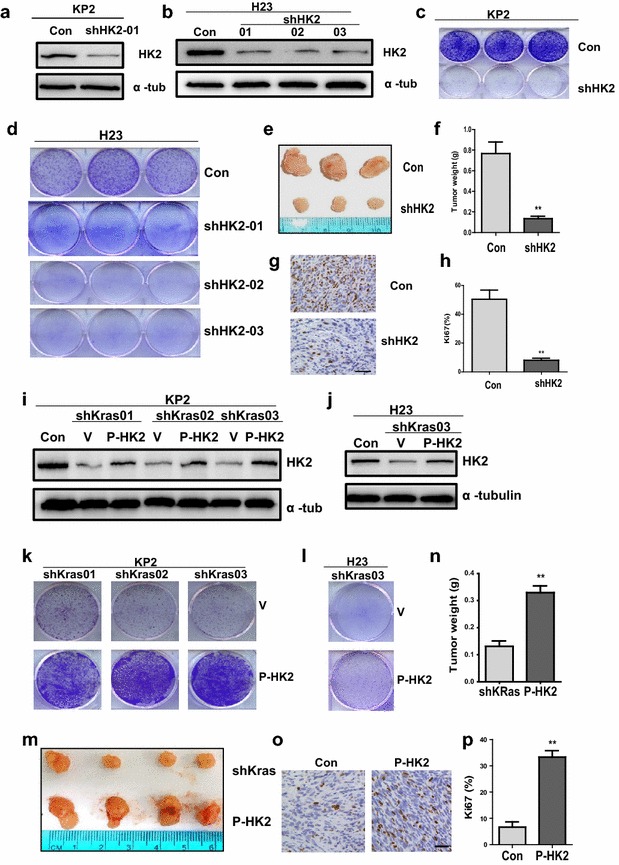
HK2 is required for Kras-driven lung tumor formation. **a**, **b** Protein level of HK2 was detected in KP2 and H23 cells expressing shRNAs for HK2 by WB. **c**, **d** Colonies were stained by crystal violet after 7 days of cell growth after knockdown of HK2 in KP2 and H23 cells. **e**–**h** Xenograft tumor growth of HK2 knockdown. Subcutaneously injected 1 × 10^6^ cells expressing scramble shRNA (CON) or shRNA for HK2 in the lower flank of NSG mice. Representative images of tumors 4 weeks after injection are shown (**e**). Quantification of tumors weight from tumors developed in NSG mice (P < 0.01) (**f**). **g** IHC staining of Ki67 from tumors developed in NSG mice carrying control or HK2-knockdown KP2 cells. **h** Quantification of Ki67 (P < 0.01) expression from representative images shown in G. **i**–**p** Rescuing HK2 assay. Protein level of HK2 was detected in three independent KRAS knockdown KP2 cell lines and one KRAS knockdown H23 cell line, all of which over-express HK2 (**i**, **j**). Then these cells were fixed and stained with crystal violet after 7 days (**k**, **l**). **m**–**p** Xenograft tumor assay of rescuing HK2 in Kras-knockdown KP2 cells. Subcutaneously injected 1 × 10^6^ KP2 cells expressing shRNA for Kras or Kras knockdown KP2 cells harboring up-regulating HK2 in the lower flank of NSG mice. Representative images of tumors at 4 weeks after injection are shown (**m**). Tumors weight (P < 0.01) from tumors developed in NSG mice (**n**). **o**, **p** IHC staining and quantitative analysis of cell proliferation marker Ki67 (P < 0.01)

To investigate whether it was required for Kras overexpression and p53 function lose-driven lung cancer cell growth, we knocked down HK2 in KP2 cells and H23 cells with three independent shRNAs: shHK2-01, shHK2-02 and shHK2-03. ShHK2-01 targeted mouse or human HK2 and caused a significant reduction of endogenous HK2 in KP2 cells and H23 cells compared to the mock cells (Fig. 2a, b). ShHK2-02 and shHK2-03 only targeted HK2 in human H23 cells and caused a significant reduction of HK2 (Fig. 2b). Knockdown of HK2 in KP2 and H23 cell lines significantly attenuated cell growth in vitro by crystal violet staining analysis (Fig. 2c, d). Furthermore, the growth of KP2 xenograft tumor cells stably expressing shHK2-1 in NSG mice was significantly suppressed compared to KP2 cells with a mock shRNA transfection (P = 0.0055) (Fig. 2e, f). As shown in Fig. 2e, f, knockdown HK2 significantly reduced xenograft tumor growth with less Ki67 expression (P = 0.0048) (Fig. 2g, h). The down-regulation of HK2 has been observed in Kras knocked down cells (Fig. 1a, b). Then we overexpressed HK2 in three independent Kras-knockdown KP2 cell lines and one Kras-knockdown H23 cell line (Fig. 2i, j). As expected, up-regulation of HK2 in the four independent Kras-knockdown lung cancer cells rescued the phenotypes that cell growth was inhibited in Kras-knockdown cell lines in vitro (Fig. 2k, l). Furthermore, the xenograft tumor growth with Kras-knockdown KP2 cells stably over-expressing HK2 in NSG mice was significantly promoted compared to Kras-knockdown KP2 cells with a scramble vitro virus (P = 0.0078) (Fig. 2m, n). The Ki67 IHC staining in xenograft tumor also showed that up-regulation of HK2 significantly increased in Kras-knockdown KP2 cells (P = 0.0066) (Fig. 2o, p). These results support that the HK2 is required for Kras-driven cell growth in vitro and tumorigenesis in vivo.

### 2-DG, HK2 inhibitor, suppresses growth of mouse and human lung cancer cells

**Fig. 3:**
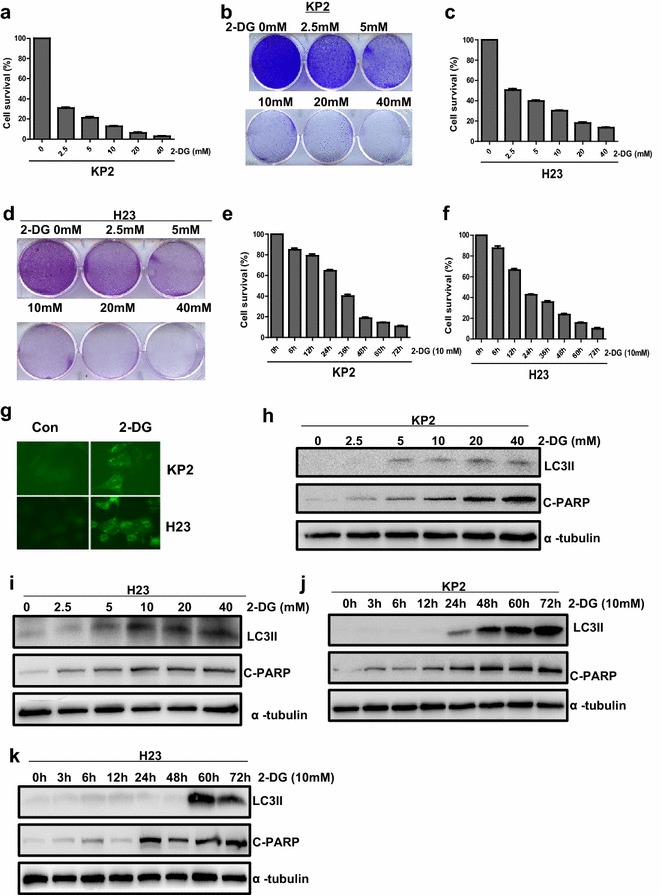
2-DG suppresses growth of Kras-driven lung cancer cells with autophagy and apoptosis pathway activation. **a**–**f** KP2 and H23 Cell growth were inhibited after treatment of 2-DG. Cell numbers were counted at 48 h after 2.5, 5, 10 and 20 mg/ml 2-DG treatment (**a**, **c**) or at 0, 12, 24, 36, 48, 60 and 72 h after 10 mg/ml 2-DG treatment (**e**, **f**). Cells treated with 2.5, 5, 10 and 20 mg/ml 2-DG at 48 h were stained with crystal violet in 6-well plate (**b**, **d**). **g**–**k** Autophagy and apoptosis pathway were activated after 2-DG treatment. **g** KP2 and H23 cells stably expressing GFP-LC3 were treated with or without 2-DG (10 mg/ml) for 48 h. **h**, **i** LC3II and Cleaved caspase3 protein level were detected by immunoblotting in KP2 cells and H23 cells treated with 2.5, 5, 10, 20 mg/ml 2-DG at 48 h. **j**, **k** Western blot detected LC3II and Cleaved caspase3 in KP2 and H23 cells treated with 10 mg/ml 2-DG at 0, 12, 24, 36, 48, 60, 72 h

**Fig. 4:**
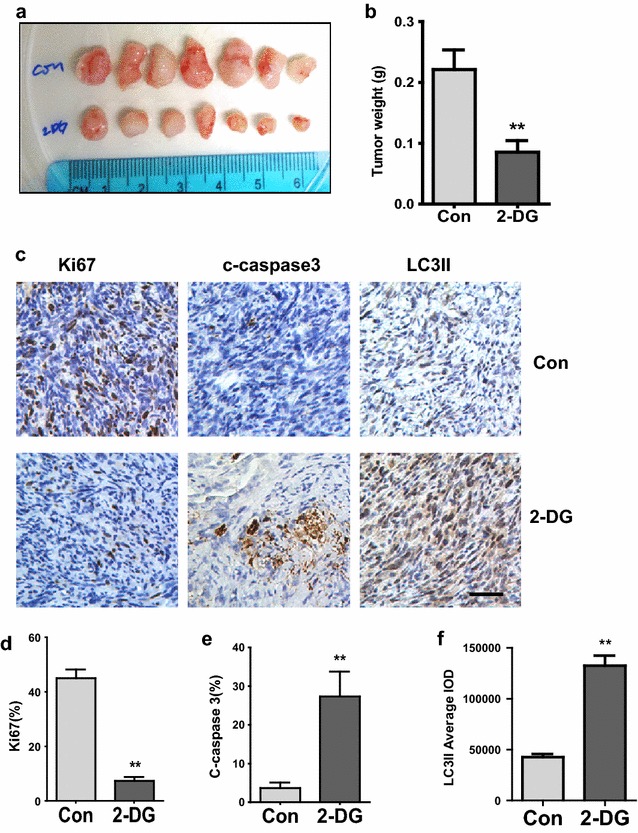
2-DG reduces tumor growth in KP2 xenograft model. **a**–**b** NSG mice were subcutaneously injected with 1 × 10^6^ KP2 cells. Once the tumor was measurable, two groups of mice were treated by I.P. injection with 800 mg/ml 2-DG or PBS for 15 consecutive days. Representative images of tumors after treatment with PBS or 2-DG are shown (**a**). Quantification of tumors weight from tumors (P < 0.01) (**b**). **c** Ki67, Cleaved Caspase-3 and LC3II were analyzed by IHC in Paraffin-embedded sections of control or treated tumor tissues. **d**–**f** Quantitative analysis of Ki67 Cleaved–caspase3 and LC3II staining in xenograft tissue (P < 0.01)

In above text, we mainly showed HK2 is essential for Kras-driven lung cancer at the genetic level. Then we test if these genetic phenotypes could be replicated by the pharmacological inhibition of HK2 enzymatic activity with 2-DG treatment. First, we examined the growth of KP2 cells and H23 lung cancer cells with 2-DG treatment. Counting cell numbers and crystal violet staining analysis were used to detect the relative growth of KP2 and H23 cells treated with 2-DG. The results indicate that cell growth is inhibited by 2-DG treatment with dose and time dependent (Fig. 3a–f). Then, KP2 and H23 cells were treated with concentration of 10 mg/ml at 12, 36 and 60 h, and DNA content was analyzed by cell flow cytometry after staining with propidium iodide. As shown in Table 1, 2-DG inhibited KP2 cell growth mainly by reducing S phase cells and significantly increasing G1 phase arrest cells, but the increased G1 phase arrest H23 cells at different time is not so much changing. The Table 1 also shows that apoptosis (sub G1) in both KP2 and H23 cells is increased stronger gradually over time. To further confirm the occurring of apoptosis, we did the western blot to check the apoptotic marker. Cleaved PARP is markedly increased in KP2 and H23 cells after 2-DG treatment (Fig. 3h–k). Also we checked autophagy pathway, LC3II expression detected by immunofluorescence and immunoblotting, as shown in Fig. 4g, 2-DG induced the increase of GFP-LC3 puncta in KP2 and H23 cells with stably expressing GFP-LC3. Then, dose and time-dependent increase of LC3-II levels were also detected by immunoblotting (Fig. 3h–k). Collectively, these data support that pharmacological inhibition of HK2-mediated glycolysis in KP2 and H23 cells might reduce cancer cell growth through inducing cell cycle arrest and activating autophagy and apoptosis pathway.

**Table 1:** Summary of cell cycle arrest patterns in two lung cancer cells induced by 2-DG

Exposure time (h)	Treatment	Cell lines							
		KP2				H23			
		G1%	%G2	S%	%Sub G1	G1%	%G2	S%	%Sub G1
12	Control	21.86	3.61	74.53	0.5	32.71	38.81	28.47	2.79
12	2-DG	44.40	0.96	54.64	0.71	31.56	48.78	19.65	3.47
36	Control	20.96	2.01	77.05	0.54	29.53	40.66	29.80	2.76
36	2-DG	70.48	0.77	28.75	2.22	34.26	48.83	16.92	4.04
60	Control	29.03	0.00	70.97	0.45	31.41	38.88	29.71	2.54
60	2-DG	87.37	7.67	4.95	4.23	39.84	43.58	16.59	8.85

### 2-DG suppresses tumor growth in vivo

Since 2-DG treatment inhibits cell growth in vitro, we predict the same efficacy of 2-DG in vivo. The NSG mice were subcutaneously injected with KP2 cells and treated with PBS or 2-DG (800 mg/kg BW) by I.P injection for 15 days. Our results revealed that tumor weights in the mice receiving 2-DG were significantly reduced in comparison with the control group (P = 0.0086) (Fig. 4a, b). The significant reduction of ki67-positive cells in 2-DG-treated mice group than in the control showed that 2-DG had inhibited KP2 cell proliferation capacity (P = 0.004) (Fig. 4c, d) in xenograft tumor sections with IHC staining. Increased cleaved caspase 3 detected by IHC in the 2-DG-treated group in comparison with the control confirmed significant induction of KP2 cell apoptosis (P = 0.0083) (Fig. 4c, e). IHC staining of LC3II in 2-DG treatment mice were also significantly induced in compared with those in the control group (P = 0.009) (Fig. 4c, f). Taken together, these studies showed that the 2-DG, HK2 inhibitor, suppresses lung cancer cell growth in vivo.

## Discussion

Many genetic and epigenetic lesions, which were detected in lung cancer, may represent potential therapeutic targets for NSCLC [24, 25]. Five common oncogenes, Kras, EGFR, ALK, ERBB2, and BRAF are represented in more than 50 % of lung adenocarcinomas [26, 27]. The oncogene Kras is the most important potential target of these genes because of its sitting at the apex of multiple growth regulatory cascades. And most of Kras overexpression patients show loss function of p53 (50–70 %) [4]. Therefore, the strategy to target Kras and p53 may be the effective therapeutic method for NSCLC. In this study, we use shRNA method to down-regulate the expression of oncogenic Kras in KP2 and H23 cells. Our results suggest that knockdown of Kras reduces cell proliferation in vitro and decreases tumorigenesis in vivo which are in consistent with previous reports in the NSCLC cell lines [28] and pancreatic cancer cell line CAPAN-1 both harboring oncogene Kras [29]. Unfortunately, the previous studies certificated that the clinical trials of Kras inhibitors, aimed at Kras C-terminal farnesylation, did not show any significantly statistical difference [30, 31] and the clinical trials of inhibitors against downstream signaling proteins RAF and MEK were also not successful [32–34]. Therefore, focusing on other new therapeutic targets or signal pathway for NSCLC may be an effective Achilles heel.

**Fig. 5:**
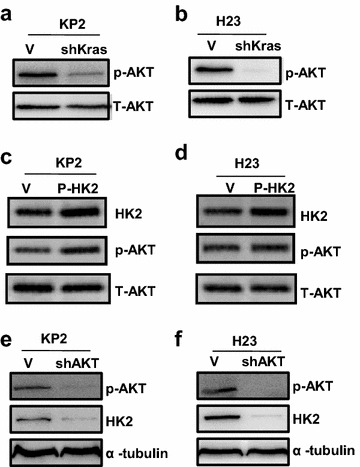
Oncogene Kras mediate HK2 expression through regulating p-AKT expression. **a**, **b** Protein level of p-AKT was detected in KP2 and H23 cells with Kras knockdown by shRNA. **c**, **d** Protein level of p-AKT and HK2 were detected in KP2 and H23 cells with overexpression of HK2. **e**, **f** Protein level of p-AKT and HK2 were detected in KP2 and H23 cells with AKT shRNA knockdown

In contrast to normal cells, tumor cells mainly depend on an increased aerobic glycolysis as the major source of ATP to fuel cell growth due to defective mitochondrial oxidative phosphorylation (termed the Warburg effect, one hallmark of cancer) [35–37]. Previous study revealed activation of oncogene Kras led to increase glycolysis [17], but the mechanism is still not clear. In this study, we found that Kras knockdown led to decrease of HK2 protein expression, as shown in Fig. 1a, b. This result support Kras increases glycolysis through regulating HK2 expression because HK2 is crucial for the Warburg effect [38]. Previous reports showed that the induction of oncogene Kras cause the AKT activation, the increase of phosphorylation at Ser473 [16]. Upon AKT activation, HK2 expression is upregulated through AKT-mediated HK2 phosphorylation [39, 40]. Mathupala et al. also reported that activation of AKT-mTOR pathway upregulates HK2 expression through induction of hypoxia-inducible factors binding to the HK2 promoter [20]. Therefore, our results confirmed knocking down the Kras in KP2 and H23 cells down-regulated AKT (Fig. 5a, b) and HK2 (Fig. 1a, b). If we overexpress the HK2 in KP2 and H23 cells, the AKT is showing a little more activation in KP2 cells (Fig. 5c) and no significant change in H23 cells (Fig. 5d). Next we knocked down AKT in KP2 and H23 cells and found down-regulated HK2 (Fig. 5e, f), which means AKT-mTOR pathway is essential in oncogene Kras mediates HK2 expression. So we revealed the key mediator of glycolysis, HK2, may be a new therapeutic target in Kras overexpression and p53 function lose-driven NSCLC.

So far, our genetic studies show that the gene HK2 is required for the growth of Kras overexpression and p53 function lose-driven lung cancer cells in vitro and in xenograft models. Upon a conditional knockdown of HK2, cancer cells growth was significantly inhibited. Moreover, Wolf et al. found HK2 knockdown by shRNAs inhibits tumor growth in a glioblastoma xenograft model [38]. Taken together, these results are consistent and reveal the HK2 involved in multiple types of carcinoma.

Given that HK2 plays the crucial role for cell growth in Kras overexpression and p53 function lose-driven lung cancer cells in vitro and in vivo, pharmacologically targeting HK2 may be explored as an effective therapy for NSCLC. 2-Deoxy-d-glucose (2-DG) as an inhibitor of HK2 is a glucose molecule and cannot undergo further glycolysis. This drug also showed promising anticancer effects in preclinical models [41]. Previous studies in vitro show that 2-DG is effective to inhibit some cancer cells growth [41, 42]. In vivo, 2-DG combining with other drugs such as paclitaxel and Adriamycin also could significantly decrease tumor proliferation in mice bearing human MV522 lung tumor carcinoma and osteosarcoma [43]. However, some later studies indicated that 2-DG treatment did not show significant antitumor activity as a single agent in vivo in some kinds of cancer cell lines [43–45]. Thus the effect of 2-DG against cancer is controversial. In our study, we choose the mouse KP2 cell (Kras mutation and p53 deletion) and human H23 cell (Kras mutation and p53 mutation) with high expression level of HK2 as research models, we found 2-DG not only inhibits KP2 and H23 cell growth in vitro, but also decreases tumor proliferation in SCID mice bearing KP2 lung cancer cells. As shown in Figs. 3 and 4, 2-DG inhibits cell growth and tumor proliferation in vitro and in vivo by inducing tumor cell apoptosis and autophagy through targeting HK2. The role of autophagy in tumorigenesis is complicated because it may represent a protective response during under some stressful conditions such as toxic stimuli, radiation, and chemotherapy [46–48], but also can limit proliferation of cells by facilitating senescence. Thus, our next study will focus on whether autophagy induced by 2-DG in Kras overexpression and p53 function lose-driven NSCLC is cytoprotective response or not cytotoxic. If 2-DG inducing autophagy is a survival response, the strategy that 2-DG combining with the inhibitor of autophagy such as CQ may be a synergistic effective therapy for NSCLC.

## Conclusions

Briefly, our genetic and pharmacological studies furthermore suggest that HK2 is one of the most important potential therapy targets and its inhibitor 2-DG is showing promising therapy efficacy in Kras overexpression and p53 function lose-driven malignant lung cancer.
