# Erratum to: Inhibition of glycolytic enzyme hexokinase II (HK2) suppresses lung tumor growth

**DOI:** 10.1186/s12935-016-0313-6

**Published:** 2016-05-24

**Authors:** Huanan Wang, Lei Wang, Yingjie Zhang, Ji Wang, Yibin Deng, Degui Lin

**Affiliations:** The Clinical Department, College of Veterinary Medicine, China Agricultural University, Beijing, 100193 China; Laboratory of Cancer Genetics, The University of Minnesota Hormel Institute, Austin, MN 55912 USA

## Erratum to: Cancer Cell Int (2016) 16:9 DOI 10.1186/s12935-016-0280-y

Following the publication of the original article [1] it was brought to our attention that there are a number of major errors within the text which were not picked up upon by the authors until after publication.

The authors accidentally copied the same pictures in Fig. 1d and Fig. 2d, Fig. 1g and Fig. 2g. The following are the correct pictures for Fig. 1d and g.

**Fig. 1:**
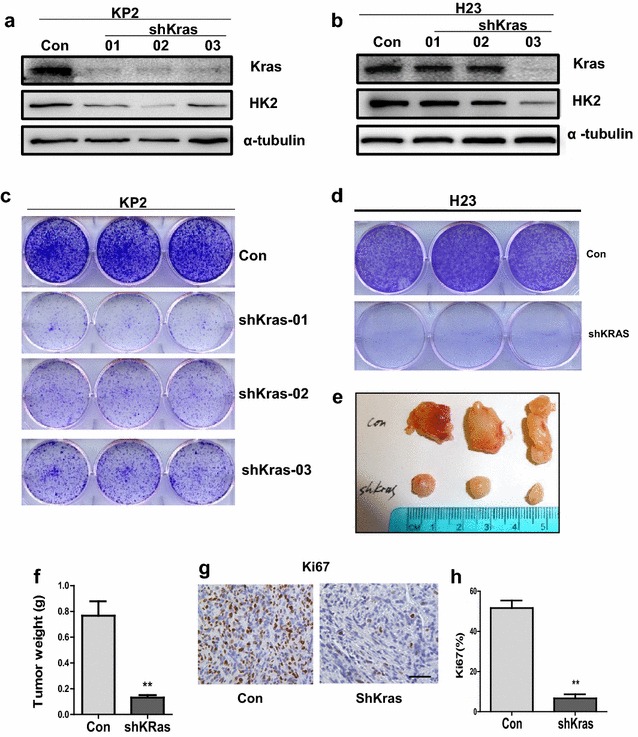
shRNA-mediated knockdown of oncogenic Kras reduces HK2 expression and suppresses growth in NSCLC cell lines. **a**, **b** Protein level of Kras and HK2 was detected in KP2 and H23 cells expressing shRNAs for Kras. **c**, **d** Clonogenic survival assays were performed to assess cell growth. Colonies were stained by crystal violet after 7 days of cell growth. **e**, **f** Xenograft tumor growth. 1 × 10^6^ cells expressing scramble shRNA (CON) or shRNA for Kras were subcutaneously injected to the lower flank of NSG mice. Representative images of tumors at 4 weeks after injection are shown (**e**). Quantification of tumors weight (P < 0.01) (**f**). **g** IHC staining of cell proliferation marker Ki67 from control or Kras-knockdown KP2 cells. **h** Quantification of Ki67 expression from representative images shown in **g** (P < 0.01)

These changes won’t affect any of the figure legends and conclusions.
